# Antiviral siRNA delivered using attenuated, anthrax toxin protects cells from the cytopathic effects of Zika virus

**DOI:** 10.1007/s11262-025-02152-4

**Published:** 2025-03-30

**Authors:** Benedita K. L. Feron, Timothy Gomez, Natalie C. Youens, Nourhan A. M. Mahmoud, Hadeer K. S. Abdelrahman, Joachim J. Bugert, Simon C. W. Richardson

**Affiliations:** 1 Exogenix Laboratory, School of Science, University of Greenwich, Central Avenue, Chatham, Kent, ME4 4TB UK; 2 Department of Biochemistry, Faculty of Pharmacy, October University for Modern Sciences and Arts (MSA), Giza, 12451 Egypt; 3 Bundeswehr Institute of Microbiology, Munich, Germany

**Keywords:** Zika, Anthrax toxin, siRNA, Anti-viral, Cytosolic delivery

## Abstract

Curative drugs are needed for the treatment of viral infections. Small interfering (si)RNA offer such a prospect but require the development of safe, effective and non-hepatotropic subcellular delivery systems. Here, 5 candidate siRNA molecules targeting defined sequences within the Zika Virus (ZIKV) genome were assayed for their ability to reduce ZIKV induced cytopathic effects in vitro. The protection of Huh-7 cells from ZIKV cytopathic effects was recorded after electroporation and the siRNA Feron-Zv2, resulting in 122.7 ± 5.3% cell viability (*n* = 3 ± standard error of the mean (SEM), 100 nM siRNA) after exposure to ZIKV relative to a virus treated control (35.2 ± 7.1% cell viability (*n* = 3 ± SEM)). Protection of BHK-21 cells was recorded after transfection with an attenuated anthrax toxin containing an RNA binding domain. Treatment with Feron-Zv4 resulted in 75.1 ± 2.9% cell viability (*n* = 3 ± SEM, 25 nM siRNA) after exposure to ZIKV. This protection was mirrored by a system containing octameric PA where a maximum of 86.2 ± 4.4% cell viability was reported (*n* = 3 ± SEM, 75 nM siRNA) after treatment with Feron-Zv2. Scrambled siRNA afforded no measurable protection. Here we report for the first time that siRNA delivered by either attenuated anthrax toxin or octamer forming ATx can protect mammalian cells from ZIKV cytopathic effects.

## Introduction

The medical use of RNA has evolved significantly since the COVID-19 pandemic. This was highlighted by 2023 Nobel Prize for Physiology or Medicine awarded to Katalin Karikó and Drew Weissman [1]. As viruses evolve, and humans encroach upon the habitats of creatures that act as reservoirs for viruses, the need for new ways to combat viral threats to human health increases. Antiviral drug development strategies have focused primarily on: (i) repurposing FDA-approved small molecules via high-throughput screens to target specific stages of the viral life cycle and disrupt viral replication; (ii) developing antibody-based therapeutics or (iii) RNA, DNA, or inactivated virus vaccines [2]. Despite significant advancements, there is still no approved vaccine against Zika Virus (ZIKV), and treatment remains palliative [2].

The use of small interfering (si)RNA to target viruses is not new. For example, siRNA molecules have been deployed to target human immunodeficiency virus, influenza virus, hepatitis B virus, human papilloma virus, West Nile virus and SARS coronavirus gene expression [3]. This siRNA technology has antiviral potential as it is highly specific, easy to design, and can be directed not only against multiple strains of the virus by targeting conserved sequence, but also at multiple sites of the viral genome simultaneously by using multiple siRNA sequences [4, 5] anticipating viral evolution.

The first siRNA drug, patisiran, was FDA approved in 2018 and has been followed by givosiran, lumasiran and inclisiran [4]. These drugs require safe and efficient delivery to the cytosol [6] and existing technologies rely on either solid lipid nanoparticle encapsulation or the use of GalNAc [6]. Balancing vector toxicity with cytosolic release has been challenging, and importantly, the body distribution of both delivery systems is limited to the liver [6]. Many strategies rooted in synthetic chemistry have been explored to try and alleviate this bottleneck. The delivery of mRNA encoding SARS-CoV-2 spike protein to striated muscle for vaccination against COVID-19 is an example of the successful use of technologies that require in situ transfection. It is of note that many other medicines that require transfection use an ex vivo transfection system exemplifying the need for a greater variety of reliable and safe in situ transfection technologies [7, 8].

One approach to overcome subcellular compartmentalisation that has gained attention over the past ten years has been the use of attenuated or recombinant protein toxins as a drug [9] or as delivery vectors [10, 11]. Previously, the cytosolic delivery of siRNA was achieved in human and primate cells using recombinant attenuated anthrax toxin (aAtx), with minimal toxicity and with efficacy comparable to that of Nucleofection® [11]. Because of the membrane interactions associated with aAtx trafficking, it was possible, through the non-covalent conjugation of siRNA, to achieve the translocation of siRNA into the cytosol of target cells [11].

**Fig. 1:**
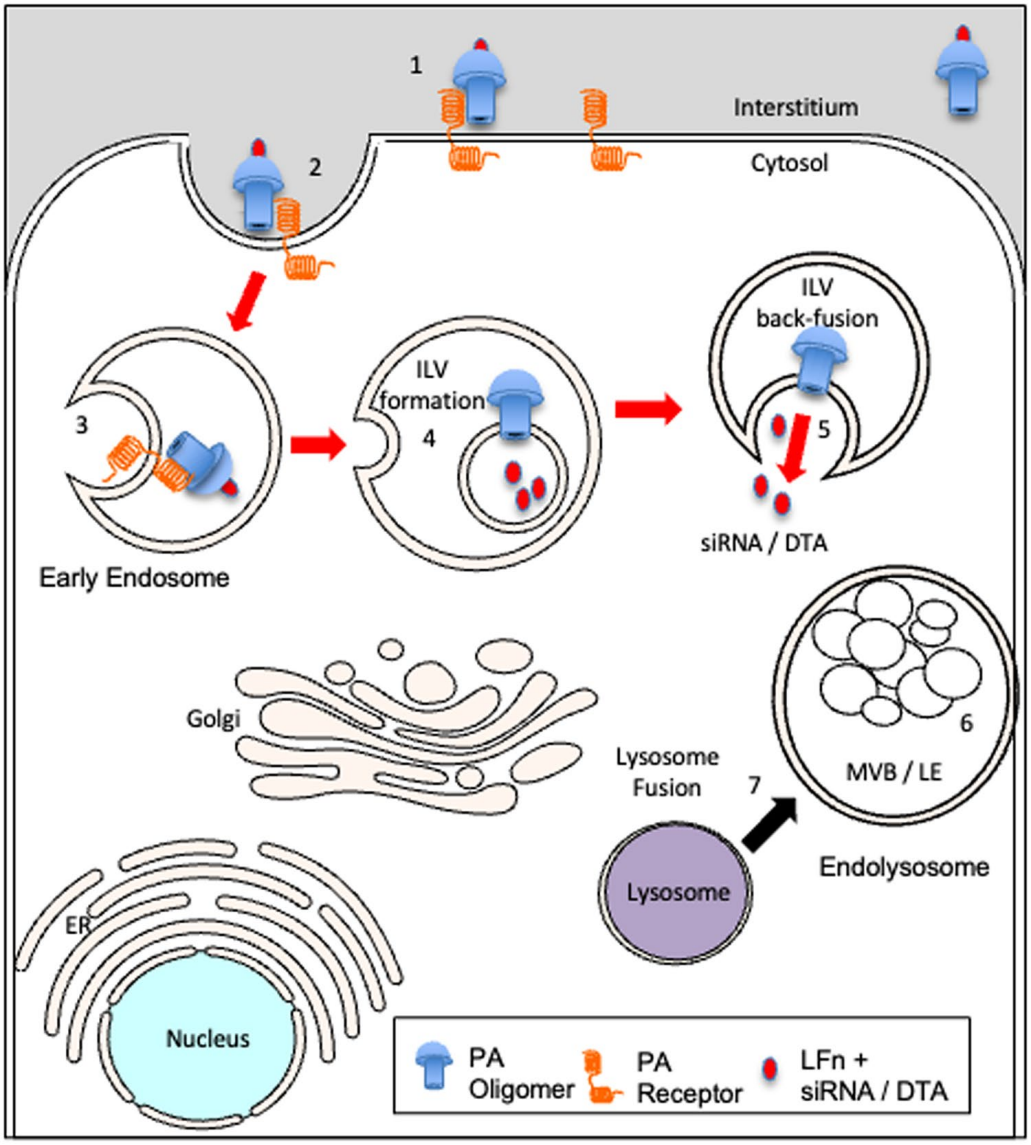
Intracellular trafficking of Atx. This cartoon depicts the intracellular trafficking of the attenuated anthrax toxin (aATx) used here to mediate the transfection of siRNA and the delivery of diphtheria toxin a chain to the cytosol. The events needed for this phenomenon are: (1) Protective Antigen (PA) receptor binding, (2) internalisation of the receptor ligand complex, (3) enrichment of the receptor ligand complex on what will become an intraluminal vesicle (ILV), (4) transmembrane translocation of LFn-PKR::siRNA or LFn-DTA driven by pH and Brownian ratcheting of the cargo through the PA pore into the lumen of the ILV and (5) the back-fusion of the ILV with the limiting membrane of the endosome, releasing luminal cargo into the cytosol. This is distinct from the default endocytic pathway that would lead to (6) digestion of luminal material in the endolysosome after (7) lysosomal fusion with the late endosome. Adapted from [11–13]

Unlike conventional transfection systems, the aATx system was not designed to rupture intracellular membranes, but rather use a nondisruptive means to deliver large molecules to the cytosol via an intermediary compartment within an endosome called an intraluminal vesicle (ILV). ATx mediates the cytosolic delivery of large molecules via a PA heptamer or octamer, being inserted into the membrane of an ILV and the ratcheting of cargo into the ILV lumen [12, 13] depicted (Fig. 1). Cytosolic delivery requires an ILV recycling (back-fusion) event, regulated by the endosomal sorting complex required for transport (ESCRT) member ALiX [12]. Previously, fluorescent cargo delivered by PA (LFn-DTA and LFn-PKR::siRNA) were observed in endosomes, the cytosol and exosomes i.e. secreted ILVs [14].

This aATx-based system has been further characterised herein, and its utility protecting cells from Zika Virus (ZIKV) cytopathic effects explored. We chose to assay the cytopathic effects of the virus (i.e. changes in ATP production) rather than monitoring gene expression by RTqPCR as we believed the data, preliminary as they are, would be less ambiguous than searching for something that may not be present i.e. ZIKV mRNA. In this instance, the cytopathic effect(s) in question were those that manifest as a reduction in ATP production i.e., ZIKV mediated cell death or detachment. This approach has been previously published in both Virus Genes and elsewhere [15, 16]. As this work focused upon cell survival rather than viral proliferation, and given the size of the siRNA:: protein complexes which could easily have been mistaken for ZIKV particles (~ 50 nm) [17] this was considered the least ambiguous way of assaying RNA interference.

**Fig. 2:**
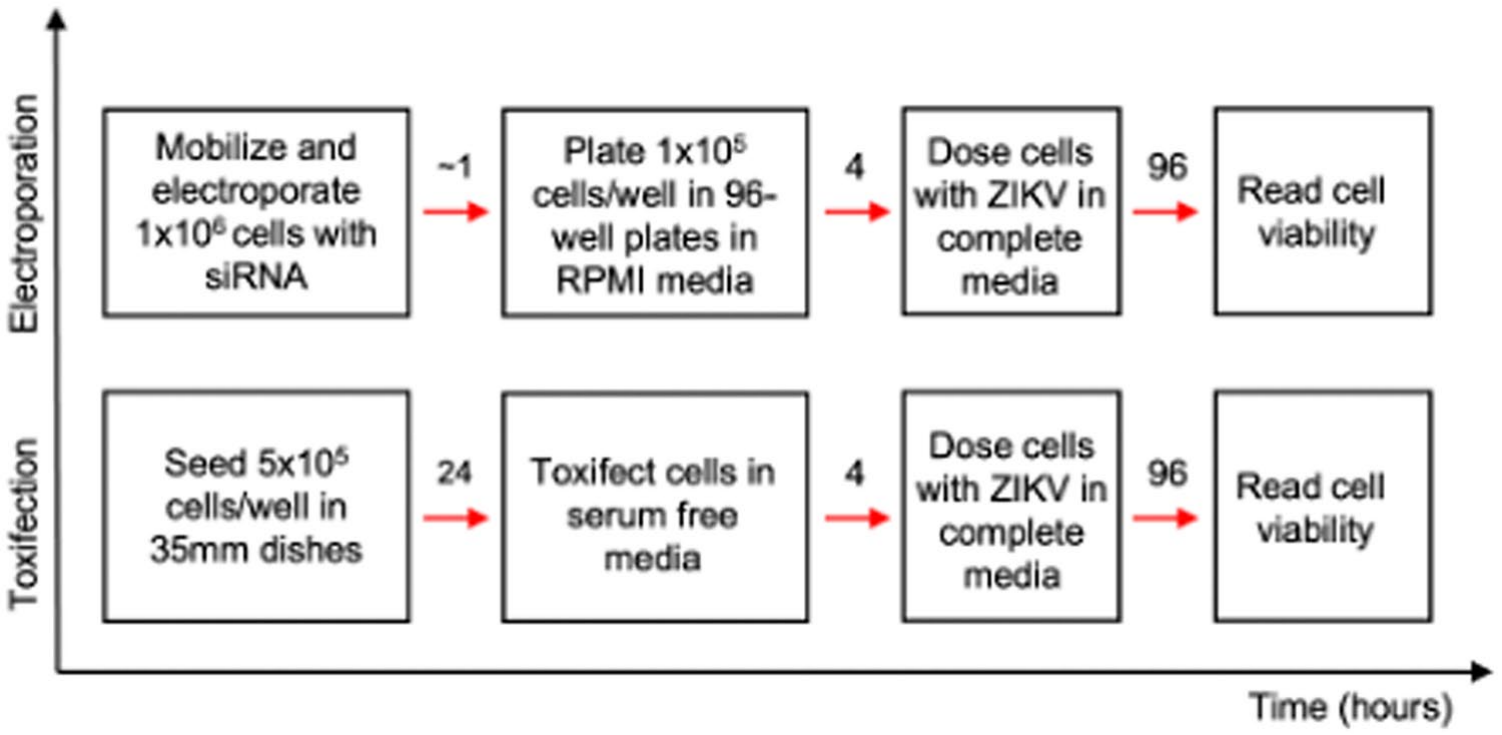
Assay for ZIKV Cytopathic Effects. This schematic diagram depicting the transfection time-courses documents the sequence of events during the testing of the siRNA listed in Table 1 using the cell lines specified. Toxifection refers to transfection using attenuated anthrax toxin

Additionally, for the first time, the use of protective antigen of 83 kDa (PA83) mutants, which complement each other to exclusively form octamers, was explored as an siRNA delivery vehicle [18] and as a way of halting the cytopathic effects of ZIKV within susceptible cells [19, 20]. We hypothesise that the siRNA trafficking efficiency of this mutant PA would be equivalent to the wild-type PA described (Fig. 1). For clarity the assays time-course is shown (Fig. 2).

## Materials and methods

All the experiments requiring the use of live virus were performed at The Bundeswehr Institute of Microbiology following local and national health and safety guidelines and using appropriate biological containment. The pET-15b LFn-DTA was a gift from John Collier (Addgene plasmid # 11,075). The production and enrichment of PA83 and LFn-PKR as well as the generation of the plasmids encoding them has been previously detailed [11].

### Cell culture

HeLa (ECCAC 93021013), Huh-7 (ECCAC 01042712) and BHK-21 (ECACC 85011433) cells were grown under standard conditions in 5% (v/v) CO_2_ at 37 °C. For culturing the Huh-7 cells, DMEM containing 10% (v/v) Foetal Bovine Serum (FBS) and 2 mM Glutamine was used and cells were split at a ratio of approximately 1:5 twice a week. BHK-21 cells were cultured in EMEM containing 2 mM Glutamine and 5% (v/v) Tryptone Phosphate Broth supplemented with 10% (v/v) FBS and split at approximately 1:6 twice a week. The HeLa cells were maintained in DMEM containing 10% (v/v) Foetal Bovine Serum (FBS) and 2 mM Glutamine, as well as penicillin and streptomycin. They were split at a ratio 1:20 twice a week. The production and characterisation of recombinant PA83 and LFn-PKR has been detailed previously [11]. Similarly, the transfection of siRNA using these reagents has also been previously reported [11]. The PA83-D^512^K/GN mutants have been previously detailed [18] and were cloned into pET151/D Topo behind MRSG and 6H tags to make comparisons with the wild-type PA previously detailed easier [11]. Transfection using PA83-D^512K^/GN::LFn-PKR::siRNA was performed using the same parameters previously described for wild-type PA83 [11]. Electroporation was performed using the Gene Pulser Xcell™ (Bio-Rad, Hertfordshire, UK) and cell viability was monitored using the CellTiter-Glo® kit (G7570 Promega, Hampshire, UK) following the manufacturer’s instructions.

### Microscopy

HeLa cells were used to seed sterile 35mm^2^ cell culture dishes (1 × 10^5^ / well) containing a sterile coverslip and maintained under normal culture conditions. The following day cells were transfected using 100 nM Cy3-labelled negative control number 1 Silencer siRNA (Cat. no. AM4621 Invitrogen, Paisley UK) using the described transfection systems according to the manufacturer’s instructions. The transfection using PA::LFn-PKR was performed as described above. After 24 h the cells were fixed using cold methanol and immunostained for LAMP 1 and 2 using clones H4A3 and H4B4 available from the Developmental Studies Hybridoma Bank (DSHB) at the University of Iowa (Iowa City, IA, USA) as previously described [11, 21]. After a 60 min hybridisation at room temperature, the coverslips were washed 3 times in PBS and a secondary anti-Mouse Texas Red conjugated antibody diluted 1:200 was incubated with the cells for an hour also at room temperature. The cover slips were washed and mounted in 50% (v/v) glycerol, PBS and 1% (w/v) *N*-propyl gallate prior to imaging using the Cy3 and Texas Red laser lines on an LSM880 confocal microscope (Carl Zeiss Ltd). Care was taken to set the detector gain for the Cy3 laser line to a level where it could be used between samples without being altered.

### Western immunoblotting

Proteins were separated using 10-well TGX gels (4–15% (w/v) acrylamide) (BioRad, Watford UK) run at 100 V for 90 min. Transfer onto 0.2µ nitrocellulose membrane was performed using a Trans-blot turbo system (BioRad, Watford UK), following the manufacturer’s instructions. Blocking was performed by incubating the post-transfer membrane with 10 mL of EveryBlot reagent (BioRad, Watford UK) prior to hybridisation at either 4 °C overnight or shaking at 37 °C for 60 min. Primary antibodies were diluted 1:1000 in EveryBlot reagent. The antibodies used were a rabbit polyclonal specific for protective antigen (Cat no. ab13808, AbCam, Cambridge UK) and a 6His specific monoclonal antibody (Cat no. 631212, Clontech, Göteborg, Sweden). Secondary antibodies were a horseradish peroxidase conjugated anti-rabbit (Sigma chemical company, Dorset UK) or an anti-mouse Starbright blue 700 antibody (BioRad, Watford UK) both used at a dilution of 1:1000 in EveryBlot reagent. Imaging was performed using Picostable ECL reagent (BioRad, Watford UK) and a ChemiDocMP imaging system (BioRad, Watford UK), following the manufacturer’s instructions. Gels were calibrated using 5µL of a Precision Plus pre-stained broad range ladder (BioRad, Watford UK).

### PA translocation assay

HeLa cells were seeded into a 96-well plate at a density of 1 × 10^4^ cells / well and left under standard incubation conditions for 24 h. The next day the media was removed and the proteins (sterile in PBS), were applied. LFn-DTA was used at final concentration of 10 µg/mL, PA83 at approximately 5 µg/mL, PA-D^512^K and PA-GN individually at 5 µg/mL and when mixed at a final PA83 concentration of 5 µg/mL.

After 1 h 100µL of complete media was added and the plates incubated for a further 71 h. After this time 20µL of 5 mg/mL MTT (Sigma Chemical Company, Dorset, UK) diluted in PBS and passed through a 0.2µ filter was added and the cells and then left under standard culture conditions for 4 h. After this time the media was removed, the excess blotted and 100µL of DMSO added. After 5 min the OD_560_ was recorded, and cell viability was calculated as a percentage of the absorbance of an untreated control. Data were expressed as Viability (% control) ± SEM at n = 3. A student’s t-test was undertaken to determine the statistical significance of the comparisons shown.

### Selection of ZIKV targeting siRNA in silico

**Fig. 3:**
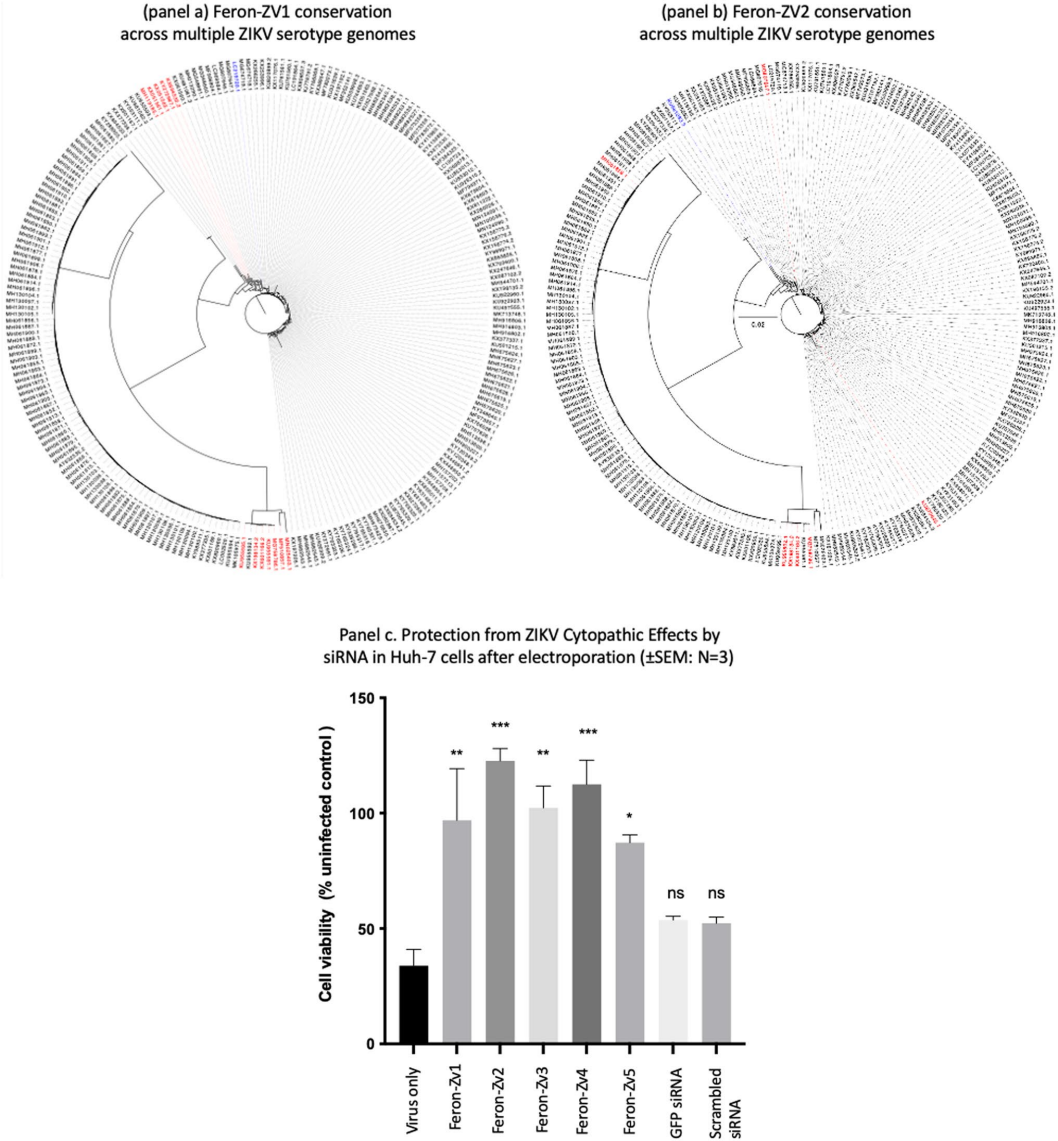
Feron-Zv1 **a** and Feron-Zv2 **b** siRNA target sequence phylogenetic conservation. Genbank accession numbers in black represent complete match of the siRNA target sequence (Table 1) with the genome documented. Accession numbers in red denote single base-pair mismatches and accession numbers in blue double base-pair mismatches. Phylogenetic trees were generated using Lasergene MegAlign Pro and FigTree Software of complete ZIKV genomes retrieved from Genbank containing no duplicates. The scale bar represents millions of years separating entries. **c** Anti-ZIKV activity after siRNA electroporation. Here the effect of Feron-Zv1-5 siRNA (100 nM) delivered by electroporation upon Huh-7 before incubation with ZIKV were documented (*n* = 3 ± SEM). Electroporation was performed using the Gene Pulser Xcell™ (Bio-Rad, Hertfordshire, UK) and cell viability was monitored using the CellTiter-Glo.® kit (G7570 Promega, Hampshire, UK). P Values denoting a statistical difference from the transfected control were: Feron-Zv1 (0.0060), Feron-Zv2 (0.0003), Feron-Zv3 (0.0031), Feron-Zv4 (0.0009) and Feron-Zv5 (0.0200)

Except for Feron-ZV5, candidates siRNA sequences were identified from published, theoretical works [22, 23] (Table 1). The sequence of Feron-Zv5 was identified using the sRNA application tab within the S-fold software (Wadsworth centre, New York State Department of Health, USA) [24] using GenBank accession number NC012532.1 corresponding to the MR-766 strain. The siRNAs listed (Table 1) were ordered as silence select siRNA molecules (Invitrogen, Paisley, UK). Where siRNA molecules were adapted from ASO sequences, the ASO reverse complement sequences were identified within the Zika reference genome, and the additional sequence required for the siRNA two base-pair 3’ overhangs identified. Phylogenetic trees (Fig. 3a & b) to assess the level of conservation of the proposed siRNA sequences were generated using Lasergene MegAlign Pro and FigTree Software from complete Zika genomes retrieved from Genbank after the removal of duplicate sequences.

**Table 1:** ZIKV siRNA targets. Details the sequence of the siRNA target and the targeted genes within the ZIKV genome

Name	Target sequence	Target	References
Feron-Zv1	AGU AUA UGA CUU UUU GG	PreM	[22]
Feron-Zv2	UUG CAU CUG CCG GAA UA	NS3	[22]
Feron-Zv3	UCA AUA UGC UAA AAC GC	C	[22]
Feron-Zv4	GUU GUA AGC ACC AAU UUU AGU	3’UTR	[23]
Feron-Zv5	CAG CUU CAG CCG UCU UCC	3’UTR	[24]

### Protection from ZIKV cytopathic effect

Cells were treated with ZIKV MR-766 (0.3 MoI) 4 h after transfection with siRNA (25-100 nM). Cell viability was monitored using the CellTiter-Glo® kit (G7570 Promega, Hampshire, UK) 96 h post-infection, following the manufacturer’s instruction. An ordinary one-way ANOVA t-test was performed followed by a Dunnet’s multiple comparisons test to calculate the statistical significance between the virus treated cells that had no siRNA (control) and the virus treated, siRNA treated cells. Each result was expressed as a percentage of the signal from the respective non-infected control. The timing of the transfection experiments is shown (Fig. 2). Additional controls were performed by treating the cells with scrambled siRNA (Cat. No. 21935200. Invitrogen, Paisley UK) (data not shown), a GFP targeting siRNA (Cat no. 12935–145, Invitrogen, Paisley UK) at 100 nM with the PA::LFn-PKR delivery system, or just the delivery system and monitoring the ZIKV cytopathic effects as before using MTT.

## Results

Here, the ability of 5 unique siRNAs (Table 1) designed to protect mammalian cells from ZIKV cytopathic effect were tested. The candidate siRNA molecules were first mapped to the NIH ZIKV reference genome and then the genome of the MR-766 strain used herein. The location and target of the siRNA molecules relative to the NIH reference genome was documented (Table 1). After excluding duplicates, the conservation of Feron-Zv1 (Fig. 3a) and Feron-Zv2 (Fig. 3b) was investigated against a selection of complete ZIKV genomes available in GenBank. As ZIKV continues to evolve, this list of serotypes is not exhaustive yet still gives an indication of the degree of conservation exhibited across the predominant ZIKV serotypes found in the wild. Here accession numbers in red denote one base pair mismatch between the siRNA sequence and the genomic sequence and accession numbers in blue denote a double base pair mismatch. The characterisation of the siRNA was performed by measuring the level of protection inferred to human hepatocyte-derived carcinoma (Huh-7) cells from ZIKV cytopathic effects after electroporation. Here all 5 siRNA (Feron-Zv1-5) molecules were tested at a concentration of 100 nM (Table 1; Fig. 3c). Feron-Zv2 afforded the most protection, yielding 122.7 ± 5.3% cell viability relative to a no treatment, infected control (33.9 ± 7.10%), followed by Feron-Zv4, which resulted in 112.5 ± 10.5% cell viability (*n* = 3 ± SEM in each instance). Feron-Zv2 targeted the ZIKV NS3 gene encoding an RNA helicase and ATPase which interacts with NS5 (which encodes the ZIKV RNA-dependent RNA polymerase (RDRP). Feron-Zv4 targeted the 3’UTR (Table 1). These results suggest that the further characterisation of all 5 siRNA sequences was warranted.

**Fig. 4:**
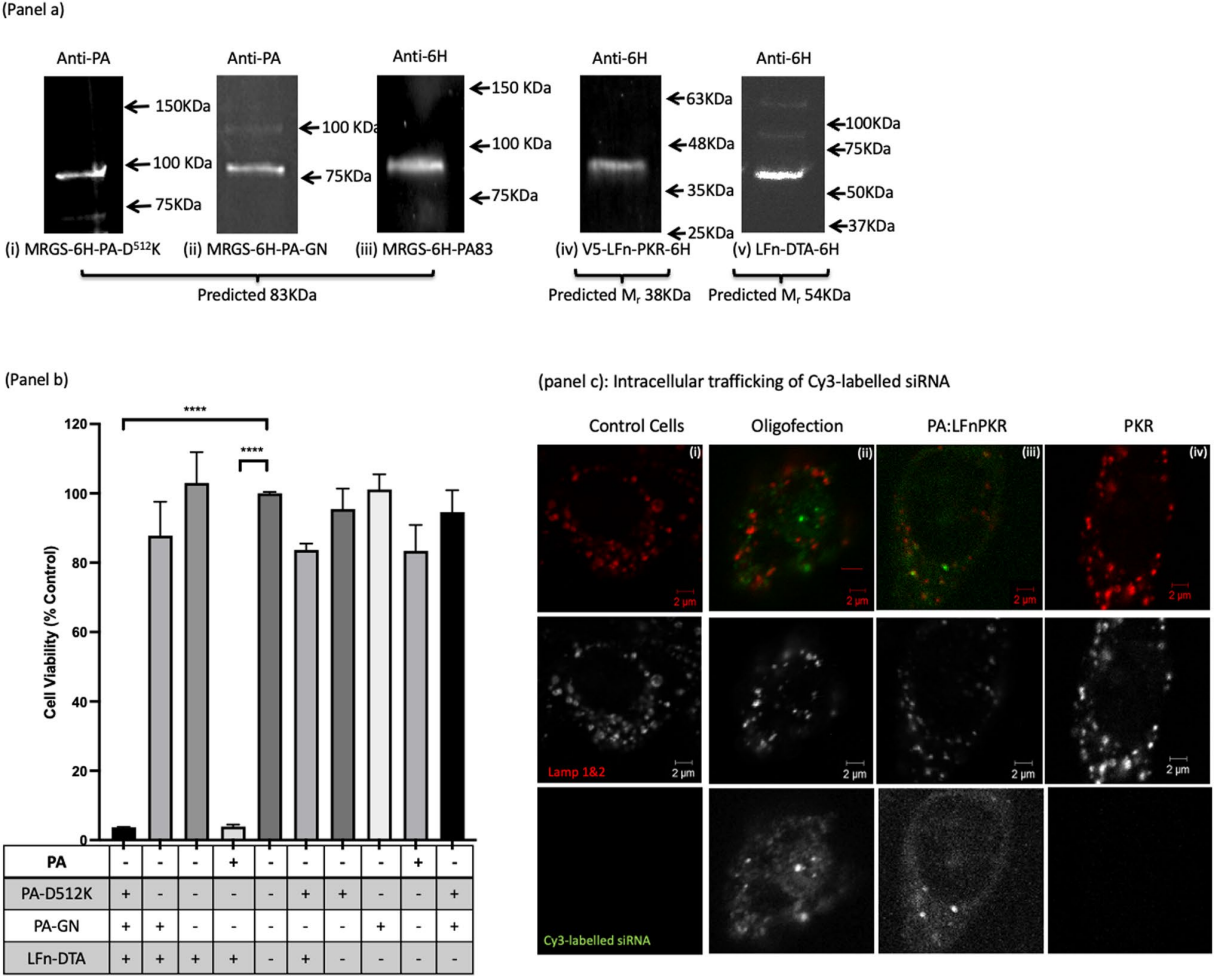
The characterisation of the recombinant proteins. **a** recorded the apparent molecular weight and immunological signature of (i) MRGS-6H-PA-D^512^K, (ii) MRGS-6H-PA-GN, (iii) MRGS-6H-PA83, (iv) V5-LFn-PKR-6H and (v) LFn-DTA-6H. Proteins were detected at the predicted molecular weight and with the predicted immunological signature. Previously the MRGS-6H-PA83 shown (ii) has been documented to be immunoreactive when probed using a polyclonal PA antibody [11]. **b**, documents translocase activity when approximately 5 µg/mL of PA (total) was incubated with 10 µg/mL of LFn-DTA-6H Panel a (v)). Here viability was assayed using MTT 72 h after the introduction of the proteins. P values were calculated using a student’s *t*-test and were < 0.0001 in each instance, when compared to an untreated control. **c** documents the translocase activity of PA using LFn-PKR::Cy3-labelled siRNA as a translocase substrate. panel c column (i) documents little detectable Cy3 signal in the control sample. Using the same detector gain settings for all images, vesicular (endosome entrapped) and nebulous (cytosolic) Cy3 signal was evident after oligofection in column (ii). Oligofection was performed using the Oligofectamine reagent (ThermoFisher) and following the manufacturers instructions. Column (iii) depicts vesicular (endosome entrapped) and nebulous (cytosolic) Cy3 signal after transfection with PA and LFn-PKR. Column iv records the effects of using only LFn-PKR and siRNA. Here when PA was omitted, no detectable vesicular or cytosolic Cy3 signal was recorded. Late endocytic structures were immunolabeled using antibodies specific for LAMP1 and 2 (from DHSB) after the cells had been subject to a cold methanol fix

As electroporation can cause cell death and does not translate readily to in vivo application, it remains unlikely that this transfection method will have clinical relevance to treating ZIKV infection. Consequently, the use of aATx (PA83: LFn-PKR) was also explored [11]. The concentration of PA83, PA83-D^512^K, PA83-GN and LFn-PKR used was 50 µg/mL (each) throughout. To this end, the proteins needed to utilise this delivery strategy, as well as those forming octameric mutants of PA83, were produced in *E. coli*, enriched and characterised (Fig. 4). Initial characterisation was by Western immunoblotting. Here octamer forming PA83 mutants were detected using a PA83-specific polyclonal antibody (Fig. 4a (i-ii)) or a 6His specific monoclonal antibody (Fig. 4: a (iii)). Some PA63 was evident however this is not unusual. Monoclonal 6His specific antibodies were also used to detect the presence of LFn-PKR and LFn-DTA and similar to the immuno-detection of the PA proteins, LFn-DTA and LFn-PKR were both evident at the predicted apparent molecular weights (Fig. 4a (iv-v)). The activity of the PA83 and mutant PA83 proteins (i.e. PA-D^512^K and PA-GN) were further characterised by examining their ability to act as translocases. As the cytosolic translocation of *Bacillus anthracis* wild-type A chains (i.e. Oedema factor (EF) and Lethal factor (LF)) remain relatively difficult to measure, the N-terminal 255 amino acids of LF fused to diphtheria toxin a chain (LFn-DTA) was used. This translocase cargo has been previously characterised as being lethal in the presence of PA83 and relatively non-toxic in the absence of PA83 [25]. Here, PA translocase activity was quantified (Fig. 4b). Again, the observed data match the predicted data and significant cell death was recorded when wild-type PA and LFn-DTA or PA-D^512^K and PA-GN and LFn-DTA were incubated together with the cells. This was in sharp contrast to the level of cell viability measured when only PA-D^512^K and LFn-DTA or PA-GN and LFn-DTA were incubated with the cells (Fig. 4: b). Further characterisation was performed by using confocal microscopy to examine the intracellular localisation of Cy3-labelled siRNA. Figure 4c contains representative micrographs recording siRNA cellular uptake. Column (i) shows the results of imaging control cells with no Cy3-labelled siRNA and no other treatment. Here, lysosome-associated membrane protein (LAMP) 1 and 2 were immunostained, denoting late endosomes and lysosomes and were visualised as being typical for the cells used [21]. Column (ii) shows the effect of oligofection of the siRNA and here Cy3-siRNA was readily visible in both late endocytic structures and as a nebulous haze within the cell (i.e. in the cytosol) denoting transfection. Similar to column (ii), column (iii) documents Cy3-labelled siRNA in vesicular structures that were both LAMP positive and negative as well as within a nebulous haze denoting cytosolic translocation. Column (iv) denotes the imaging of an LFn-PKR::Cy3 siRNA control (i.e. no PA). Here little Cy3 signal was documented denoting reduced levels of uptake in the absence of PA. These data complement and confirm those previously published [11]. Care was taken to maintain a constant gain setting when acquiring these images. The detector gain settings for both channels were set using the oligofection control and the same settings were used to image for all the other samples.

In all instances, cell viability was expressed as a percentage of treated, uninfected cells to control for treatment toxicity. ZIKV strain MR-766 (0.3MoI) was used as it had been previously characterised by the investigators, remains clinically relevant, and was readily available to handle at an appropriate containment level. To deliver these siRNA agents to the target cytosolic compartment, an experimental system utilising attenuated anthrax toxin was used [11]. As there was protection from the cytopathic effects of the virus after siRNA electroporation, the ability of wild type and a mutant PA (a component of the attenuated anthrax toxin) that could only form octamers [18, 26] rather than predominantly heptamers was also investigated. This investigation was to ascertain any significant improvement in the performance of this system when using the mutant PA relative to the wild-type PA.

**Fig. 5:**
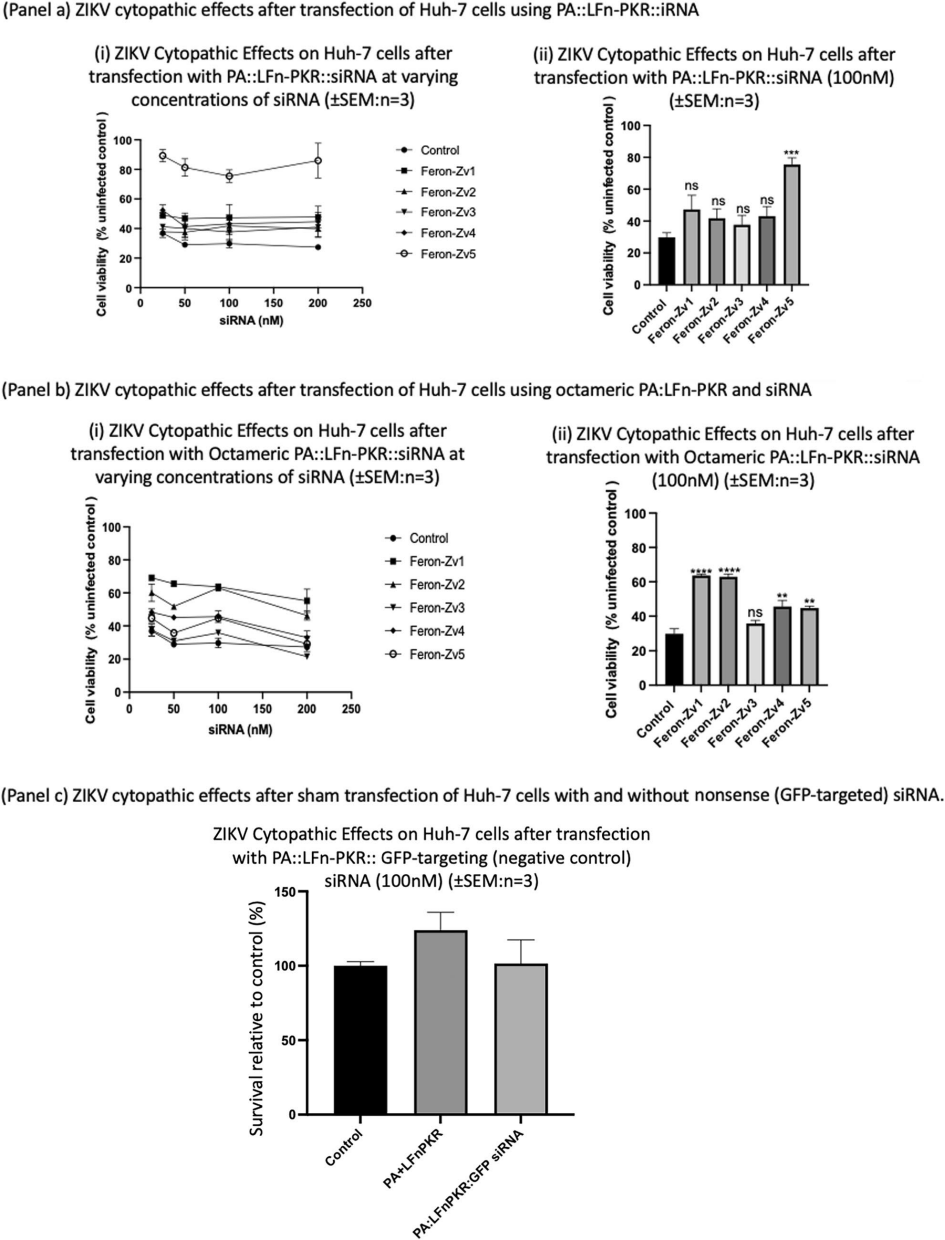
**a** RNA interference mediated protection from ZIKV cytopathic effects in Huh-7 cells. Here the cytopathic effects of ZIKV upon Huh-7 cells after transfection using PA::LFn-PKR::siRNA were recorded. When transfection was undertaken immediately prior to incubation with the virus, only Feron-Zv5 has a protective effect (*P* value 0.0007; *n* = 3 ± SEM). This effect is exemplified (ii) when a snapshot of a 100 nM siRNA concentration was examined. **b** RNA interference mediated protection from ZIKV cytopathic effects in Huh-7 cells using octameric PA mutants. panel b records the effect of using octameric PA mutants MRSG-6H-PA D^512^K and MRSG-6H-PA-GN as part of the transfection system. Although the protective effect of Feron-Zv5 is less profound, statistically significant protection was documented for Feron-Zv1 (*P* value < 0.0001), -Zv2 (*P* value < 0.0001), -Zv4 (P value 0.001) and -Zv5 (P value 0.0016) siRNAs (*n* = 3 ± SEM). **c** Sequence specificity control in Huh-7 cells. Here the effect of control siRNA upon the Huh-7 cell cytopathic effects after exposure to ZIKV. Here no deviation of the level of effect measured in the control population i.e. only ZIKA virus treatment is evident. This indicates that the effects observed (Figure; panel 5 a & b) were sequence specific

With the exception of Feron-Zv5, Human (Huh-7) liver cells (Fig. 5a) showed little benefit from incubation with both ZIKV and siRNAs Feron Zv1-4 using the heptameric delivery system (Fig. 5a). However, as Feron Zv5 showed significant protection against the cytopathic effects of ZIKV, the ability of the forced octameric PA mutants to deliver siRNA was investigated. When the octameric mutants were used to deliver the siRNA, all except Feron Zv3 showed significant protection with Feron-Zv1 and Feron-Zv2 offering ~ 40% more protection than the virus-only treated control cells (p > 0.0001 in each in-stance) (Fig. 5b).

Figure 5c documents a negative control, testing for sequence specificity in Huh-7 cells. No protection was recorded above that of untreated cells exposed to ZIKV when either PA and LFn-PKR or PA and LFn-PKR hybridised with an siRNA specific for GFP, serving as a nonsense control.

This experiment was then repeated using another cell line susceptible to ZIKV cytopathic effects i.e. BHK-21.

**Fig. 6:**
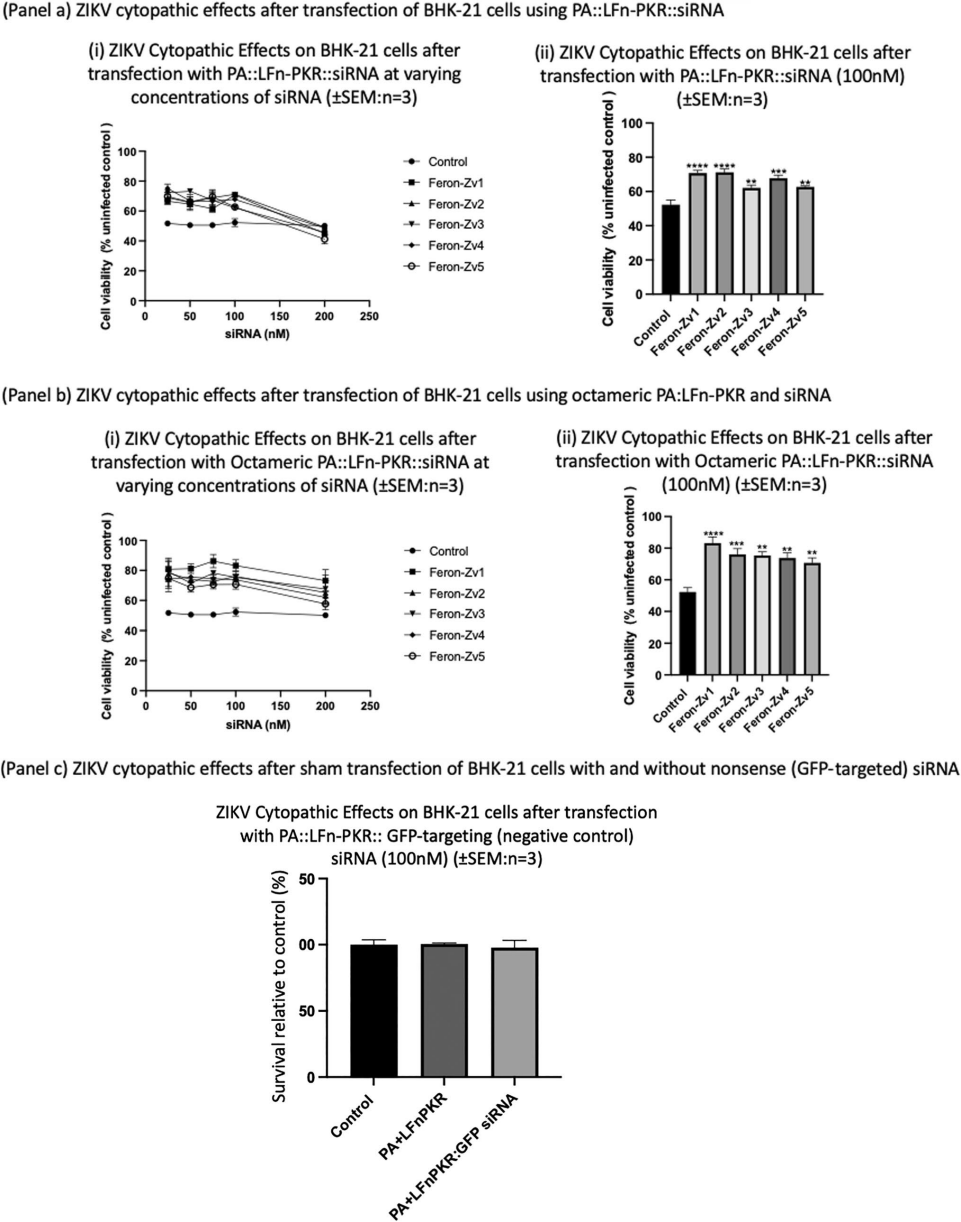
**a** RNA interference mediated protection from ZIKV cytopathic effects in BHK-21 cells. Feron-Zv siRNA (25-100 nM) delivered by PA83-D^512^K/PA83-GN, and LFn-PKR protected BHK-21 cells from ZIKV cytopathic effects, *n* = 3 ± SEM. (i) Feron-Zv siRNA (75 nM) delivered by PA83-D^512^K/PA83-GN, and LFn-PKR protects BHK-21 cells from ZIKV cytopathic effects, (*n* = 3 ± SEM). An ordinary one-way ANOVA calculated p values to be **b** (ii): Feron-Zv-1 (< 0.0001), Feron Zv-2 (< 0.0001), Feron Zv-3 (0.0091), Feron Zv-4 (0.002), and for Feron Zv-5 (0.0060). **b** RNA interference mediated protection from ZIKV cytopathic effects in BHK-21 cells using octameric PA mutants. Octameric PA mutants were also able to deliver siRNA and again in each instance protection was documented, (*n* = 3 ± SEM). An ordinary one-way ANOVA calculated p values to be (from left to right) for Feron-Zv1 (> 0.0001), Feron-Zv2 (0.0009), Feron-Zv3 (0.0011), Feron-Zv4 (0.0020), and Feron-Zv5 (0.0066). **c** reports the effect of control siRNA upon the BHK-21 cell cytopathic effects after exposure to ZIKV. Here no deviation of the level of effect measured in the control population i.e. only ZIKA virus treatment was evident. This indicates that the effects observed (panel a and b) were sequence specific

All 5 siRNAs protected BHK-21 cells from ZIKV cytopathic effects over a range of concentrations (25-100 nM) when combined with the predominantly heptameric PA83 and LFn-PKR (Fig. 6a). Maximum overall protection was documented when 25 nM siRNA was used (Fig. 6a). Cell viability was increased relative to the data from Huh-7 cells (Fig. 5a) for cells treated with Feron-Zv1 (66.6 ± 2.3%), Feron-Zv2 (68.9 ± 2.0%), Feron-Zv3 (72.3 ± 1.7%), Feron-Zv4 (75.1 ± 2.9%) and Feron-Zv5 (69.7 ± 2.8%) (*n* = 3 ± SEM in all instances). The infected untreated control yielded 50.8 ± 4.0% cell viability relative to the uninfected untreated control (100% cell viability). In this instance Feron-Zv1 and 2 afforded the most protection.

Octameric PA mutants PA-D^512^K and PA-GN combined with LFn-PKR and siRNAs [11, 18] also protected BHK-21 cells from ZIKV cytopathic effects over a range of concentrations (Fig. 6b). Maximum overall protection was documented after 75 nM siRNA was used (Fig. 6b). Cell viability was increased for cells treated with Feron-Zv1 (86.2 ± 4.4%), Feron-Zv2 (72.7 ± 2.7%), Feron-Zv3 (78.2 ± 0.9%), Feron-Zv4 (74.5 ± 2.3%) and Feron-Zv5 (70.6 ± 2.9%). The infected untreated control yielded 50.7 ± 2.4% cell viability (again *n* = 3 ± SEM in all instances). In this instance Feron-Zv1, targeting preM, afforded the most protection. One of the experimental objectives associated with the use of the forced octamer mutants was to assess any obvious increase in delivery efficiency. From the data reported here (Fig. 5a–b) and Fig. 6a–b), it remains difficult to say if the octameric mutants were more effective than wild-type PA with any certainty, though the gains that these mutants may afford could be cell type specific, allowing the transfection of cells that are less amenable to transfection with wild-type PA (Fig. 6b). Figure 6c is a repeat of the sequence specificity control (Fig. 5c) in BHK-21 cells. Please note the concentrations of siRNA reported for the BHK-21 and Huh-7 cells are different, with BHK-21 cells being subject to higher doses than the Huh-7 cells.

## Discussion

The persistence of viral outbreaks, the effects of climate change and new routes of transmission highlight ZIKV as a global threat to public health [1]. Vaccine development has been ongoing and includes nucleic acid vaccines, inactivated attenuated vaccines, and viral-vector vaccines [2], and although several vaccines have been shown to be effective in animals, none have been approved as a medicine [27]. Previously siRNA has been used to reduce inflammation associated with ZIKV infection [28] with siRNA transfection via solid lipid nanoparticles, and silencing the pre-inflammatory CXCL12 gene. An alternative approach has been to use siRNA to induce ZIKV resistance in *Aedes aegypti* with the aim of preventing transmission [29]. A strategy, similar to the one adopted herein has been to silence elements of the viral genome, specifically targeting the 3’ untranslated region (3’-UTR) [30]. This strategy was shown to reduce viral replication using RTq-PCR in C6/36 cells. Rabies virus glycoprotein derived peptide (RVG) -engineered extracellular vesicles loaded with ZIKV-specific siRNA prevented vertical transmission of ZIKV in pregnant mice [31]. In this instance four different siRNA sequence were evaluated targeting NS4A, PrM, NS1 and NS3 ZIKV genes and these siRNA were shown to cross both the placental and the blood brain barriers when combined with the recombinant ECV delivery system.

Here, novel siRNA was shown to provide prophylactic protection when delivered by electroporation or using engineered attenuated anthrax toxin in vitro. It can be assumed that both electroporation, the use of wild-type heptamer forming PA and the octameric mutant PA proteins, in conjunction with LFn-PKR effected the intracellular delivery of siRNA in mammalian cells, which enabled the targeting of ZIKV mRNA, increasing post-infection cell viability relative to the virus-only treated cells (Fig. 3c, Fig. 5 and Fig. 6). This conjecture was further supported by the lethality of LFn-DTA in conjunction with both the octamer and heptamer forming PA (Fig. 4b) [25].

The selection of cells used in these studies was based on the susceptibility of liver and kidney cells to ZIKV infection [19, 20]. These results suggest that Huh-7 cells are more susceptible to ZIKV cytopathic effects than BHK-21 cells, providing a better cell model giving a readout that is relatively easy to interpret i.e. the cells are alive or dead. Further work is required to evaluate the effectiveness of siRNA as a treatment post-infection, and to investigate the efficacy of these systems in more complex models, like human brain organoids or even in vivo. Here a reduction in ATP production was favoured over attempting to measure any reduction in virus particle production as the PA multimer (~ 14 nM in length) [11, 13] bound to LFn-PKR which dimerises to bind siRNA, and thus has the capacity to interact with the alpha clamp on two PA oligomers remains compounded by the fact that one PA oligomer can bind 3 LFn molecules [13], would be of a similar size to the ZIKV particle and as a consequence light scattering would not be readily able to discern the difference between the two, whilst it may have been possible to measure a difference between toxin::siRNA complexes and virus particle using small angle neutron scattering [11], the logistics of this would not be trivial due to the containment needed for the safe handling of the virus.

Considering the activity and biodistribution of wild-type ATx in mice [32], there is reason to believe that aATx may navigate the endomembrane system to deliver siRNA to the cytosol of cells in vivo, providing the kinetics of delivery are fast enough to negate the barrier that serum RNAse enzymes represent. As the modification of siRNA and the use of a readily available cell receptor (i.e. the asialoglycoprotein receptor) have been shown to be clinically useful, these data warrant a further investigation in vivo. Given the overexpression of the anthrax I receptor TEM8 within neoplastic tissue, utility may also be found treating neoplasic mass resulting from viral infection as is seen with non-ZIKV infections such as those related to Human Papilloma Virus. This supposition is further supported by work showing the transfection of HeLa cells using PA, LFn-PKR and LFn-GAL4 delivering siRNA and antisense oligonucleotides, respectively [11].

Previously, the octameric PA mutants have been characterised as having what appears to be a phenylalanine-clamp with a slightly larger diameter than wild-type PA [18]. Herein it was conjectured that this may have an impact upon the translocation efficiency associated with the aATx siRNA delivery system, given that the phenylalanine -clamp for wild-type Atx has been reported to be 0.6 nm [13] which, if static, would be too small for an siRNA molecule to traverse. It is possible that any advantage this affords was negated by a reduction in movement across the PA articulating surfaces, however before any firm conclusion can be drawn more evidence is required. Equivalently these data may also point to phenylalanine-clamp transit not being a rate limiting step during siRNA translocation over the PA translocase.

In addition to the questions that remain unanswered in this paper, and aside from the question of in vivo activity, there remains the possibility of administering siRNA after the inoculation of the cells with the virus. At what time point might the siRNA become ineffective at not only negating cytopathic effects but also preventing the production of more virus?

Finally, the effect of multiple scrambled control siRNA upon ZIKV cytopathic effects was investigated (Figs. 5c, 6c), and supported by the variation of the effects seen from the 5 different siRNA molecules all with different sequences, it remains likely that the effects reported denoted sequence specificity. Here, the variation in sequence between Feron ZV-1–5 adds an additional control for sequence specificity and off-target effects. Combined treatment with multiple siRNAs targeting different regions of the ZIKV genome may also enhance silencing and improve cell protection in relation to viral evolution.

Clearly more work is needed to meet the need of patients who have contracted ZIKV, and the results of this investigation into a medicine. This is a very long journey, with many opportunities for failure. However, we eagerly anticipate undertaking experiments to establish the body distribution of both the siRNA and the siRNA::aATx delivery system and measuring any anti-viral activity as well as establishing a safety profile measuring stability and any toxicity evident after a dose escalation study in a small rodent model. However, these experiments are beyond the scope of this paper.

## Conclusions

Here preliminary data characterises 5 novel siRNA sequences have reduced the cytopathic effects of ZIKV in specific, ZIKV susceptible cells. Additionally, it was shown that these siRNA molecules could be successfully delivered using modified, attenuated anthrax toxin comprised of either heptamer or forced octamer forming PA83.

## Patents

WO2014203008 and WO2020030923 were granted to the University of Greenwich and are currently under evaluation for licensing by Vitarka Therapeutics Ltd. This patent protects various transfection methods utilizing attenuated anthrax toxin.

## Data Availability

Raw data will be made available upon the request of the referees and will be made available generally on request once published.
